# Long-term effects of protraction facemask combined with fixed appliance therapy in severe skeletal Class III adolescent: A case report with 5-year follow-up

**DOI:** 10.1016/j.ijscr.2024.110306

**Published:** 2024-09-24

**Authors:** Nan Zhou, Zhengxian Zhu, Liting Jiang, Xuechao Wang, Niansong Ye

**Affiliations:** a Shanghai Huaguang Dental Clinic, Shanghai, China; b Department of Orthodontics, Shanghai Xuhui District Dental Center, Shanghai, China

**Keywords:** Protraction facemask, Skeletal Class III adolescent, Long-term effect

## Abstract

**Introduction and importance:**

The decision between orthodontic camouflage therapy and orthodontic-orthognathic surgical treatment for developing skeletal Class III malocclusion presents a significant challenge for orthodontists.

**Case presentation:**

This case report describes the camouflage treatment of a severe skeletal Class III adolescent at the post-pubertal stage.

**Clinical discussion:**

Protraction facemask combined with a bonded acrylic splint expander was initially used to correct the developing skeletal Class III malocclusion. Then the patient received fixed appliance therapy. The duration of active treatment was 14 months. Anterior crossbite was corrected, along with stable occlusion and harmonious facial condition. The results remain stable at the 5-year follow-up period.

**Conclusion:**

Treatment with protraction facemask followed by fixed appliance therapy was possibly effective in a long-term observation, even in skeletal Class III adolescent at the post-pubertal stage.

## Highlights

• Protraction facemask was presented to be useful in the treatment of skeletal Class III adolescent at the post-pubertal stage.
• Long-term stability was achieved in this case report.
• Clockwise rotation of the mandibular plane was achieved and the facial proportion of the patient was improved.

## 1 Introduction

Maxillary expansion combined with a protraction facemask has been used with frequency for the correction of developing Class III children [1]. However, treatment options for skeletal Class III malocclusion in adolescents after pubertal growth remain controversial. The decision between orthodontic camouflage therapy and orthodontic-orthognathic surgical treatment presents a significant challenge for orthodontists, particularly when a nonsurgical approach is preferred.

The effects of orthopedic facial mask treatment in the early mixed dentition of Class III patients with maxillary deficiency have been well documented in earlier reports. These effects can be summarized as a combination of maxillary advancement, clockwise rotation of the mandible and tipping of the lower incisors. Comparative studies have underscored the critical importance of optimal treatment timing for skeletal Class III malocclusion, given the complex combination of skeletal and dental components [[2], [3], [4]]. Many adolescents in the post-pubertal stage opt to delay treatment, waiting for future orthognathic surgery. Despite challenges in predicting orthopedic changes in older developing patients, the effect of facemask in altering mandibular growth direction has been acknowledged, with a significant dentoalveolar response observed.

This case report presents the use of protraction facemask combined with a bonded acrylic splint expander to correct a skeletal Class III adolescent at the post-pubertal stage. The treatment resulted in clinically acceptable and aesthetically pleasing outcomes. A 5-year follow-up was conducted to assess the long-term stability.

## 2 Diagnosis and etiology

A 13-year-old girl complained of anterior crossbite. Accompanied by her parents, she was referred for an evaluation of orthodontic treatment. The patient exhibited no chronic systemic diseases and was in good general health.

**Fig. 1:**
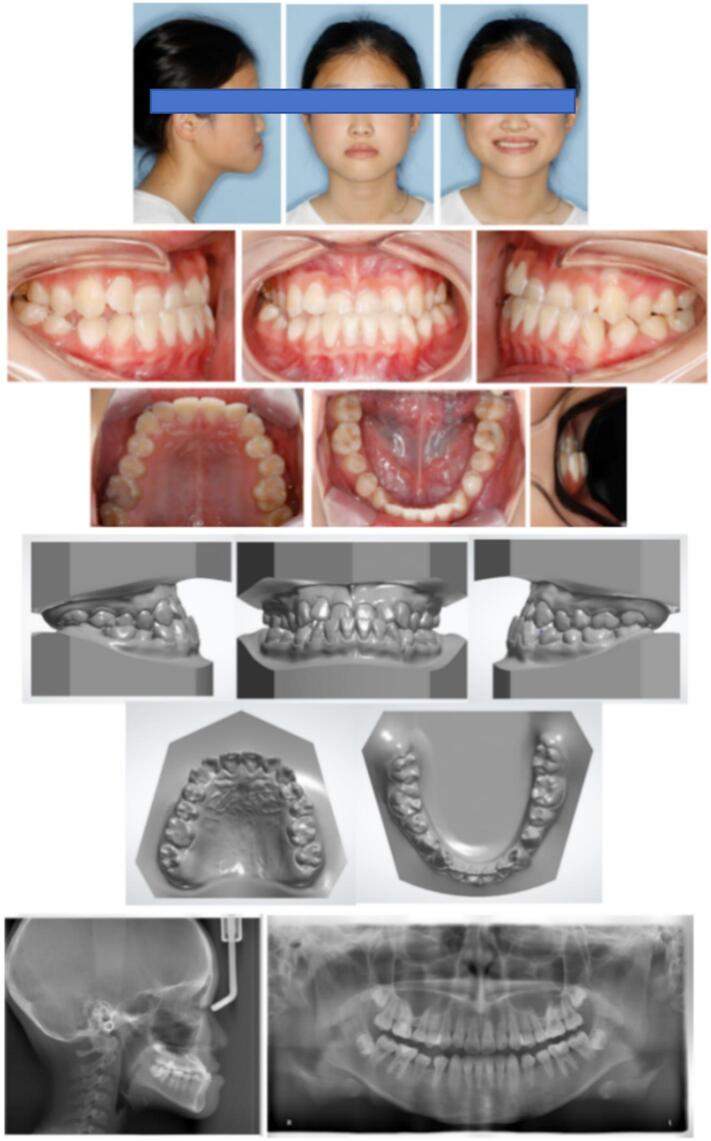
Pre-treatment facial and intraoral photographs, pre-treatment dental casts and pre-treatment radiographs.

Extraoral examination revealed a concave, anteriorly divergent facial profile. Mild facial asymmetry was observed, with the right cheek appearing slightly plumper than the left. Vertical facial proportions were within the normal range. Temporomandibular examination revealed the absence of symptoms such as joint noise or pain. However, a functional shift was observed when her mandible was guided into centric relation (Fig. 1).

Intraorally, a Class III molar relationship was noted bilaterally. The patient had anterior crossbites and posterior crossbites on the maxillary right first and second premolars. The overjet was −3 mm and the overbite was −2 mm. The maxillary arch showed mild crowding and the curve of Spee was accentuated. Her maxillary dental midline was coincident with the facial midline, but there was slight deviation of the mandibular dental midline to the left. Additionally, four dental caries filled with resin composite were detected (Fig. 1).

The panoramic radiograph revealed the presence of four third molars. The lateral cephalometric analysis indicated a skeletal Class III relationship (ANB, −5.1°) due to mandibular skeletal excess (SNB, 88.2°), with a maxillary skeletal deficiency (SNA, 83.1°) and a hypodivergent growth pattern (FMA, 21.2°; SN-MP, 26.2°). The jaw discrepancy resulted in midface retrusion. To compensate for an underlying skeletal disproportion, the maxillary incisors were proclined (U1-SN, 116.4°), and the mandibular incisors were retroclined (IMPA, 83.8°). Cervical vertebral maturation evaluation revealed a fifth cervical stage (CS5), indicating low anticipation of craniofacial growth. The patient's mother also had a skeletal III pattern, which appeared to be the heredity factor (Fig. 1) (Table 1).

**Table 1:** Cephalometric measurements.

Measurement	Pretreatment	Posttreatment	Norm
SNA (°)	83.1	84.7	83.0 ± 4
SNB (°)	88.2	86.8	80.0 ± 4
ANB (°)	−5.1	−2.1	3.0 ± 2
Wits (mm)	−8.5	−3.8	−1.0 ± 0
SN-MP (°)	26.2	28.7	33.0 ± 4
FMA (°)	21.2	22.8	28 ± 4
U1-NA (mm)	6.3	9.0	4.0 ± 2
U1-SN (°)	116.4	125.3	105.7 ± 6.3
L1-NB (mm)	2.9	2.0	6.0 ± 2
IMPA (°)	83.8	79.9	96.5 ± 7.1
U1/L1 (°)	134.7	126.0	127.0 ± 9
UL-EP (mm)	−3.1	−1.2	2.0 ± 2
LL-EP (mm)	2.1	−0.2	3.0 ± 2

## 3 Treatment objectives

The following treatment objectives were included: (1) improving the skeletal Class III relationship, (2) correcting anterior and posterior crossbites, (3) obtaining normal overjet and overbite, (4) establishing stable functional occlusion, (5) leveling the curve of Spee, (6) improving facial profile.

## 4 Treatment alternatives

All records were thoroughly reviewed with the patient and her parents. Orthognathic surgeries combined with orthodontic treatment were first discussed. Waiting until cessation of growth, then the patient can be treated with a LeFort I maxillary osteotomy to advance the maxilla and bilateral sagittal split ramus osteotomy (BSSRO) to set back the mandible. A maximum facial outcome might have resulted from this approach.

The second option involved camouflage treatment using orthodontics alone. Considering the patient still had some growth potential, conventional orthopedic therapy using a protraction facemask could be attempted. Palatal expansion could help to correct posterior crossbites and improve the maxillary protraction effect. Class III elastics would be used to obtain distalization of the mandible and lingual inclination of the mandibular incisors. However, limited improvements would occur in the facial harmony with the traditional therapy. In addition, more time and high patient compliance were demanded during the camouflage treatment.

The patient and her parents were provided with a detailed explanation of the risk/benefit considerations associated with both treatment options. Despite the availability of orthognathic surgery combined with orthodontic treatment, the family expressed a strong preference for the less-invasive camouflage treatment. It was emphasized that, due to the unpredictable nature of the Class III growth, there might be a potential need for additional treatment including orthognathic surgery in the future.

## 5 Treatment progress

**Fig. 2:**
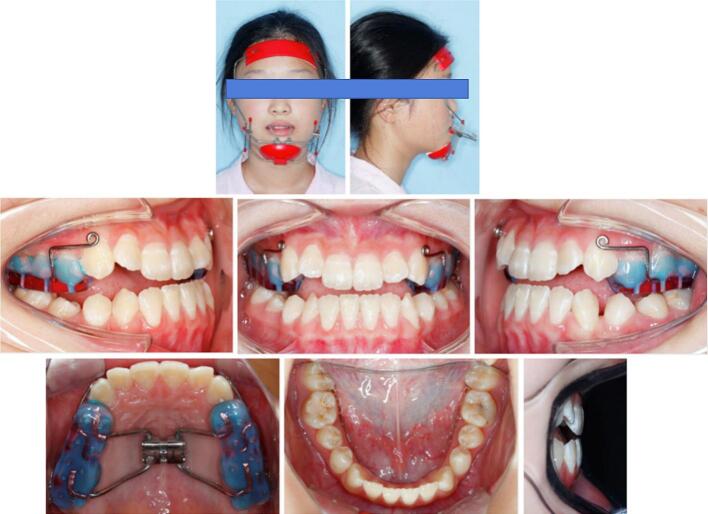
Initial treatment using protraction facemask combined with a bonded acrylic splint expander.

Treatment began with bonding an acrylic splint expander on the maxillary posterior teeth. The expansion screw was activated twice every day until over correction of transverse discrepancy was achieved. Meanwhile, the patient was provided with a protraction facemask which delivered 500 g force with extraoral elastics placed on each side (Fig. 2).

**Fig. 3:**
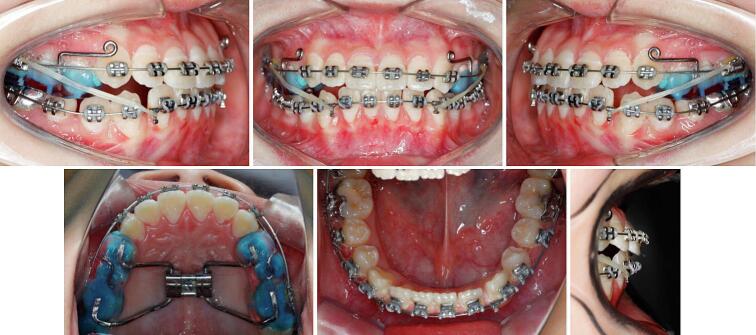
The 6-month time point during the treatment.

**Fig. 4:**
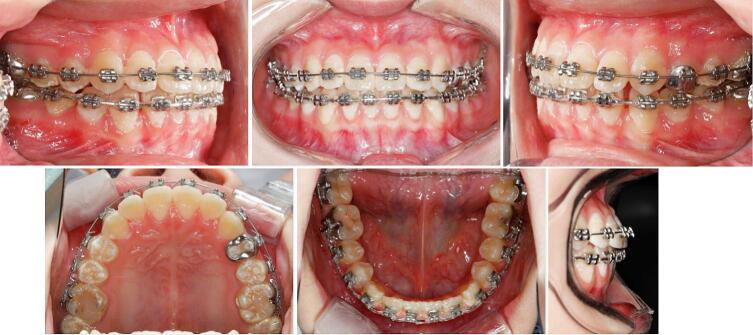
The 11-month time point during the treatment.

Nearly one month later, a positive overjet was obtained and 0.022 ∗ 0.028-in slot brackets were bonded to the maxillary teeth. After initial alignment and leveling, nickel-titanium rectangular archwires were placed. At 6-month point, the patient's facial profile was apparently improved, then the protraction facemask was removed. Class III elastics were engaged with maxillary rectangular archwires (Fig. 3). Final detailing of the occlusion was accomplished with 0.018 ∗ 0.025-in stainless steel archwires (Fig. 4).

Total treatment time was approximately 14 months. After the orthodontic treatment, the patient was instructed to wear removable thermoplastic retainers specially designed for Class III elastics, intended to maintain the correction achieved. The work has been reported in line with the SCARE criteria [5].

## 6 Treatment results

**Fig. 5:**
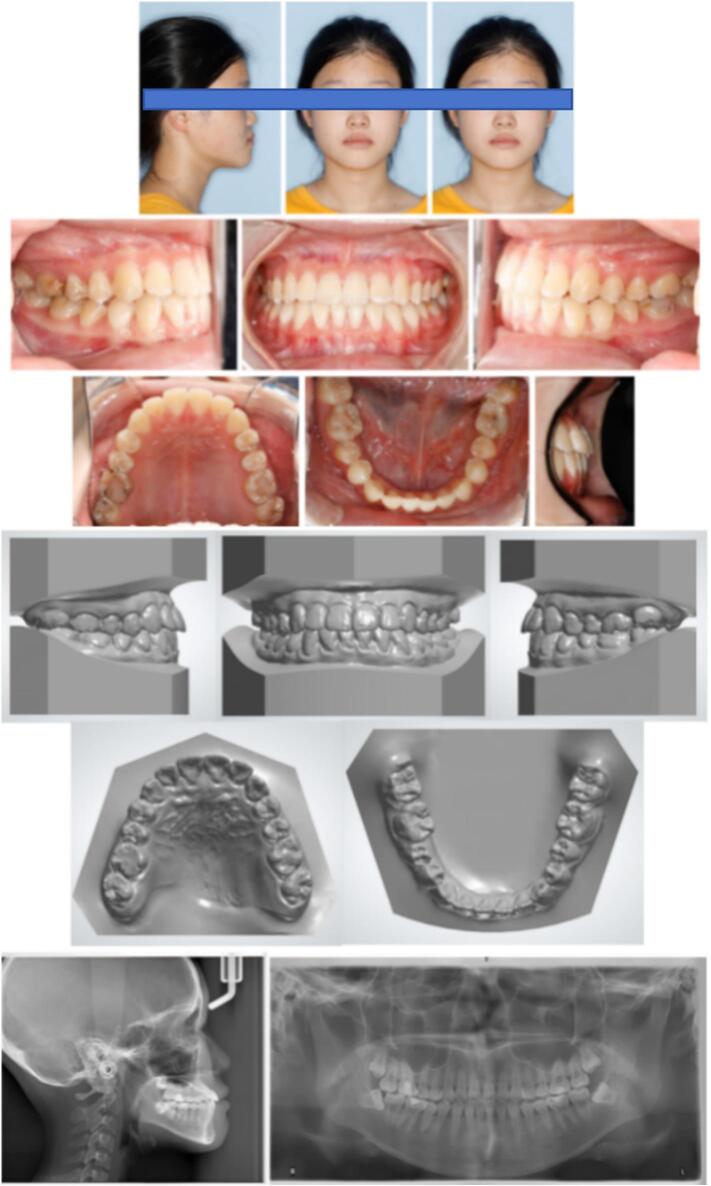
Post-treatment facial and intraoral photographs, post-treatment dental casts and post-treatment radiographs.

The post-treatment records confirmed the achievement of all treatment objectives. Anterior and unilateral posterior crossbites were successfully corrected along with stable occlusion. Dental midlines basically coincide with her facial midline. Significant improvement in facial concavity was observed and the functional shift was eliminated. No temporomandibular joint pain was reported following orthodontic treatment (Fig. 5).

In the post-treatment panoramic image, root parallelism was deemed acceptable, with no significant bone loss or root resorption observed. The lateral cephalometric analysis revealed a skeletal change in maxilla forward movement (SNA, from 83.1° to 84.7°). ANB changes (ANB, from −5.1° to −2.1°) can be attributed to the anterior growth of the maxilla and the clockwise rotation of the mandible (FMA, from 21.2°–22.8°), resulting in an improved facial profile (Fig. 5) (Table 1).

**Fig. 6:**
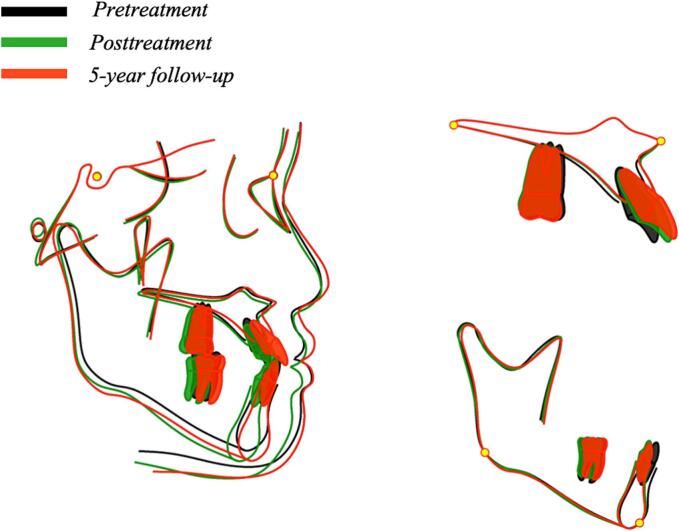
Superimposed cephalometric tracings and profile.

The superimposition of cranial base indicated that the maxillary incisors were proclined labially, and the mandibular incisors were retroclined. The maxillary molars were extruded due to the use of Class III elastics. Additionally, the position of the upper lip was closer to the esthetic plane (UL-EP, from −3.1 mm to −1.2 mm) (Fig. 6).

Five years after treatment, stable occlusion and harmonious facial condition were maintained, with no recurrence of the anterior crossbite. Comparison of the post-treatment and 5-year follow-up cephalograms showed small changes. Since the patient is now 18 years old and unlikely to experience significant growth, no further big changes are expected (Fig. 6). The patient and her parents expressed satisfaction with the treatment outcome.

## 7 Discussion

Clinicians have been trying to discuss the selection between camouflage or surgical orthodontic treatment for skeletal Class III adults [[6], [7], [8]]. However, the optimal treatment approach for developing adolescents after pubertal growth remains a topic of debate. Many older children with skeletal Class III malocclusion opt to delay treatment until growth cessation before making a final decision. Currently, the precise point at which interventions for Class III patients become ineffective remains unknown.

Protraction facemask is commonly used to treat developing Class III children and adolescents with maxillary deficiency. This case report describes the use of a bonded palatal expansion appliance in conjunction with the protraction facemask to promote the orthopedic advancement of the maxilla. Heavy-force elastics were employed to connect bilateral hooks on the bonded expander to the curved rod of the facemask, generating a forward and downward traction force on the maxilla. The protraction facemask system can be adjusted by tightening or loosening screws set within the appliance. It is recommended that the angle between the direction of the elastic pull and the Frankfort horizontal plane be less than 30 degrees.

Most studies indicate that orthopedic modification for developing Class III patients is typically initiated during early developmental phase, such as early mixed or late deciduous dentition. Franchi et al. suggested that assessing bone age using cervical vertebral radiographs could be helpful to determine the optimal timing for maxillary protraction [3]. They found that protraction facemask is most effective when initiated during the prepubertal stage in cervical vertebral maturation (CVMS I). However, Patrick reported that older children aged 10–14 years still benefit from the protraction therapy, but to a lesser degree [2]. Gregory even proposed the possibility of treating adult Class III malocclusion with maxillary expansion and protraction facemask [9].

In our patient, the lateral cephalometric analysis indicated a post-pubertal stage in cervical vertebral maturation (CVMS V). The pretreatment skeletal discrepancy (ANB, −5.1°; Wits appraisal, −8.5 mm) originated from her hypoplastic maxilla and prognathic mandible. Familial environmental factors may play a substantial role in the etiology of her Class III malocclusion. Considering the post-pubertal stage in this patient, maxillary expansion combined with protraction therapy was employed. The benefits of palatal expansion have been extensively discussed by clinicians, including its potential to increase arch width, loosen or activate circummaxillary sutures and initiate movement of the maxillary complex [1]. In our patient, a significant skeletal change in maxilla forward movement was observed, resulting in a marked improvement in the facial profile. These findings suggest that orthopedic modification for developing Class III patients may still be beneficial even in the post-pubertal stage, particularly in cases of mild to moderate skeletal discrepancy.

In our patient, the use of Class III elastics contributed to the extrusion of the mandibular incisors and maxillary molars. Consequently, there was an increase in lower anterior facial height, which may not be ideal for hyperdivergent Class III patients. Based on the original vertical facial pattern, our patient belongs to the low-angle (SN-MP, 26.2°). The slight increase in her lower anterior facial height resulting from the clockwise rotation of the mandibular plane ultimately improved the facial proportions.

Several scientific studies have investigated the long-term effects of early camouflage orthodontic treatment for Class III malocclusion. Ngan et al. found that only 70 % of patients treated with facemask in a clinical trial maintained a positive overjet during the 4-year follow-up [10]. The physiological relapse due to mandibular growth indicated the importance of maintenance and long-term follow-up. To maintain the positive outcomes of previous orthopedic treatment with a facemask throughout the growing period, various appliances are anticipated to be used. FR-3 appliance, a chin cup and retainers equipped with Class III elastics are commonly considered effective in maintaining the achieved anteroposterior correction [11]. Our patient was monitored until the age of 18 to ensure treatment stability. Gender differences in Class III malocclusion suggest that male patients may require a longer observation period compared to females [12].

Pretreatment factors related to the stability of early Class III treatment have been identified in various studies. Inoue et al. concluded that the most influential factor was the horizontal distance between molars in centric relationship [13]. Other factors, such as mandibular length, Wits measurement, and gonial angle may also have a slight influence on stability. In a clinical trial conducted by Tahmina et al., adolescent patients with Class III malocclusion were divided into two groups: stable and unstable [14]. The unstable group was found to have a significantly larger gonial angle. Additionally, Lee reported that skeletal Class III patients featuring lingually positioned lower incisors tend to exhibit more favorable long-term outcomes after facemask therapy [15].

With microimplants as skeletal anchorage, bone anchored maxillary protraction and micro-implant assisted rapid palatal expansion (MARPE) became more effective and increased the scope of orthopedic growth modification [16,17]. Lee investigated the long-term effects of facemask therapy using skeletal anchorage and found it showed a greater advancement of maxilla compared with tooth-borne anchorage [18]. In the future, therapy with skeletal anchorage may become increasingly prevalent among skeletal Class III adolescent patients in the post-pubertal growth phase.

## 8 Conclusion

Treatment with protraction facemask followed by fixed appliance therapy was possibly effective in a long-term observation, even in skeletal Class III adolescent at the post-pubertal stage.

## Consent

Written informed consent was obtained from the patient's parents/legal guardian for publication and accompanying images. A copy of the written consent is available for review by the Editor-in-Chief of this journal on request.

## Ethical approval

Our institution does not require ethical approval when reporting individual case. This case report does not contain any research involving human or animals. Written informed consent has been obtained from the patient. The name of our institution is Shanghai Huaguang Dental Clinic.

## Funding

No funding.

## Author contribution

Nan Zhou and Zhengxian Zhu contributed to treating the case and writing the case report. Liting Jiang and Xuechao Wang contributed to literature review and treating the case. Niansong Ye contributed to the critical management of the case report and reviewing the case.

## Guarantor

Niansong Ye is the guarantor.

## Research registration number

Our institution does not require registration of research studies when reporting individual case. This case report does not contain any research involving human or animals. The name of our institution is Shanghai Huaguang Dental Clinic.

## Declaration of competing interest

No conflict of interest.
